# Zebrafish small molecule screens: Taking the phenotypic plunge

**DOI:** 10.1016/j.csbj.2016.09.001

**Published:** 2016-09-18

**Authors:** Charles H. Williams, Charles C. Hong

**Affiliations:** a Department of Cell and Developmental Biology, Vanderbilt University School of Medicine, Nashville, TN 37232, USA; b Department of Medicine, Vanderbilt University School of Medicine, Nashville, TN 37232, USA; c Department of Pharmacology, Vanderbilt University School of Medicine, Nashville, TN 37232, USA; d Vanderbilt Institute of Chemical Biology, Vanderbilt University School of Medicine, Nashville, TN 37232, USA; e Research Medicine, Veterans Affairs Tennessee Valley Healthcare System, Nashville, TN 37212, USA

**Keywords:** High-throughput screening, Whole-organism screening, Phenotypic screening, Phenome-wide association study

## Abstract

Target based chemical screens are a mainstay of modern drug discovery, but the effectiveness of this reductionist approach is being questioned in light of declines in pharmaceutical R & D efficiency. In recent years, phenotypic screens have gained increasing acceptance as a complementary/alternative approach to early drug discovery. We discuss the various model organisms used in phenotypic screens, with particular focus on zebrafish, which has emerged as a leading model of *in vivo* phenotypic screens. Additionally, we anticipate therapeutic opportunities, particularly in orphan disease space, in the context of rapid advances in human Mendelian genetics, electronic health record (EHR)-enabled genome–phenome associations, and genome editing.

## 1 Introduction

For much of human history, therapies for various ailments came about from astute phenotypic observations and serendipity [31]. For instance, the origins of digoxin, a cardiac glycoside currently in use for heart failure, can be traced directly to a traditional herbal remedy for dropsy made from the foxglove plant [37]. With the advent of modern biochemistry and molecular biology, drug discovery became dependent on the target-based approach to systematically screen for thousands and even millions of agents that modulate a particular biological target chosen based on a rational therapeutic hypothesis. In the decades that followed, an unprecedented number of new therapeutics have transformed modern medicine and pharmaceutical industry [21]. However, despite the disproportionate focus and funding on target based approaches for the past two decades, the pharmaceutical industry as a whole delivered fewer “first-in-class” drugs using this approach than using a phenotypic approach [56]. In fact, the cost, and the risks, of developing a new pharmaceutical entity have skyrocketed in the recent decades, with the costs of developing a new drug seeming to grow exponentially, a trend termed “Eroom's Law”, to contrast with the Moore's Law describing exponential growth in computing power [51]. There are a number of reasons for this alarming decline in efficiency of pharmaceutical development. Obvious reasons include unforeseen off-target effects and toxic metabolites that result in deleterious effects in humans. While late stage failure in clinical trials captures headlines, a key reason for the sustained decline in productivity may lie in the earliest stages of drug discovery: specifically, poor target selection. For an industry grown around target-based discovery, picking a wrong target based on an invalid therapeutic hypothesis can be a death knell, a situation made worse by the fact that consequences might not be apparent until significant expenditure of time and effort. There are numerous causes of poor target selection, but chief among them appears to inadequate insight into human pathophysiology provided by *in vitro* and preclinical models [2], [70].

Given the pitfalls of target-based screening, phenotypic screening has reemerged as an attractive alternative and complementary approach to drug discovery. As the name implies, this approach focuses on phenotypic perturbations – observable changes in complex biological function caused by small molecules – to identify chemical modulators of physiological or disease processes in a target-agnostic manner. The observed phenotype results from integration of all cellular pathway perturbations in the context of an active biological system, be it an individual cell or an entire organism. A phenotypic screen, by definition, identifies chemotypes that affect a biologically meaningful target or targets, including key nodes responsible for integrating cell pathways and behaviors. Importantly, since a phenotypic screen is conducted without regard to *a priori* knowledge of targets, it has the potential to discover new therapeutic targets, which may have greater impact at the systems level than established targets. Moreover, in contrast to target-based screens, a phenotypic screen permits discovery of compounds that affect a desired outcome *via* engaging multiple targets in a synergistic manner that may not have been otherwise anticipated. Indeed, recent studies have shown that polypharmacology is not necessarily deleterious, and that engagement of multiple targets can sometime be more effective for treatment of certain disease [50]. While a knowledge of the precise pharmacological target is traditionally considered essential, although not required by the FDA, to push a drug development forward; there is increasing willingness to be target agnostic provided there is a compelling biological rationale and an unmet medical need [32].

In contrast to traditional observational approaches, which were low-throughput and therefore depended on serendipity, the modern phenotypic screen combines the advantages of phenotype-based approaches with the latest high-throughput chemical screening capabilities. In this review, we will provide a brief overview of various models used in phenotypic screens, with a focus on zebrafish based screens, which has emerged as a powerful *in vivo* model amenable to high-throughput and high-content analyses, and a look to the future of phenotypic screening.

## 2 Phenotypic screening modalities

Modalities of phenotypic screens can be broken into two components: the biological model and the assay outputs. These two factors must be considered prior to any screen. A number of model systems have been used in phenotypic screening, ranging from single cells, to organoids and whole organisms.

Cell based screens vary in scope of potential readouts from a simple cell viability assay to complex cell behavior analyses. At the simple end of the spectrum, most screens for potential anti-cancer agents are cell viability assays using established cancer cell lines [53]. At the complex end, Lum and colleagues have screened small molecules in HCT116 human colorectal cancer cells using multiplexed luciferase assays and dot blotting to monitor multiple pathways simultaneously [25]. By assessing multiple pathways in a quantitative manner, they were able to collapse the cellular phenotypes elicited by individual compounds into a “fingerprint.” Traditionally, determining mechanism of action (MOA) can be laborious, however; such an approach provides mechanistic insights by clustering compound induced “fingerprints” to those obtained from an siRNA library [24]. Cell based screens have also been conducted in an image based analytics paradigm. Peppard and colleagues identified novel autophagy regulators in HeLa cells expressing LC3 (microtubule-associated protein light chain3)-GFP (green fluorescent protein) fusion protein as an autophagy readout. LC3 is normally cytosolic, however during autophagy is recruited to autophagosomal membranes, which manifest as GFP granules in this read out. When nutrient starved cells are treated with lysomotropic agent hydroxychloroquine (HC), which inhibits the lysosome, LC3-GFP degradation by autophagy is blocked. Using HCS imager *Incell 3000*, a 250,000 compound screen was conducted to identify inhibitors of the formation of autophagosomes, which was thresholded as *< 4 GFP granules*[42] Notably, the authors validated this assay with wortmannin, a known inhibitor of autophagosome formation and used this as a positive control to set the threshold.

While most cell based screens have been conducted in established cell lines grown in simple monolayers or suspension, investigators have developed 3-D organoid models of tumor cells, with the aim of developing an *in vitro* model that is more relevant to human tumor biology, including the role of metabolically quiescent tumor stem cells and the effect of hypoxia gradient within solid tumors. For instance, Walsh and colleagues have developed a model of spheroids derived from primary human tumors, utilizing intrinsic fluorescence properties of FAD and NADH called optical metabolic imaging (OMI). OMI has previously been shown to serve as an early endpoint biomarker for drug response [60]. Using this technique the authors carried out a screen for small molecules that altered metabolic activity of tumor spheroids [61].

In the past few years, human induced pluripotent stem cells (hiPSCs) have emerged as a promising human biological platform for phenotypic screening. Since their initial description less than a decade ago, researchers have created iPSC models of a myriad of human diseases using patient-derived iPSCs [58]. For example, Burkhardt and colleagues have generated hiPSC from ALS patients and demonstrated that neurons differentiated from these hiPSCs exhibit TDP-43 aggregation, a pathological hallmark of ALS. Using an image-based screen based on TDP-43 aggregation in neurons generated from ALS hiPSCs, they discovered that known small molecule inhibitors of the Na^+^/K^+^ ATPase, GSK3 and CDK could ameliorate this phenotype, providing supporting not only for prior studies that have implicated these proteins as potential ALS therapeutic targets but also the use of patient-derived iPSCs for drug discovery [4].

Cell based screens, while providing an inexpensive, quantitative and high throughput platform for phenotypic screening, suffer from several disadvantages. Despite advances in engineered tissue constructs, cultured cells do not exist in a native biological context and lack critical tissue interactions and paracrine factors which clearly play an important role *in vivo*. Compound liabilities such as poor metabolic stability, suboptimal bioavailability and undesirable off-target as well as on-target effects are not recognized early on during the primary screen. Such issues can be addressed from the start with *in vivo* chemical screening of living organisms and whole animals. Thus far, large-scale *in vivo* phenotypic screens have been conducted in various model multicellular organisms ranging from nematode such as *C. elegans* to vertebrates such as zebrafish.

**Fig. 1:**
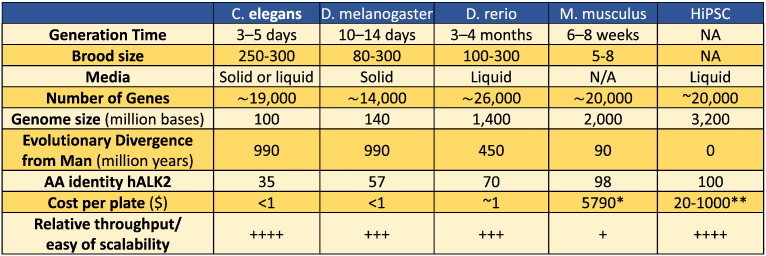
Comparison of model organisms used in phenotypic screens. Commonly accepted numbers for generation time and brood size are listed, along with media for animal maintenance, evolutionary divergence, gene number and genome size [29]. Also listed, the evolutionary divergence from man and the amino acid sequence identity to the human BMP receptor ALK2 (hALK2). Unit cost: approximate cost of animals needed to screen a 96-well plate of compound libraries, in triplicate. *For mice, this is the approximate cost to purchase 288 mice from Jackson Labs. **Cost of iPSC varies significantly depending on differentiated cell type, culture methods and screening conditions. Relative throughput/ease of scalability: ++++, very high (close to *in vitro* HTS); +++, high (up to tens of thousands compounds/week); +, low (up to hundreds of compounds/week).

For instance, Petraschek and colleagues have performed a small molecule screen for compounds that affect aging in the nematode. From this screen, they identified 60 compounds that increase *C. elegans* lifespan without obvious deleterious effects. Concordant with existing genetic models of aging, over half of the hit compounds increased the animal's resistance to oxidative stress [71]. Importantly, this screen revealed a large number of candidate targets that are conserved in humans and hence represent potential therapeutic targets to slow aging. Of course, *C. elegans* is still a very simple animal organism, with a rudimentary physiology, lacking for instance discrete circulatory system. Moreover, *C. elegans* has a very short life cycle (approximately 3.5 days) and each adult hermaphrodite has precisely 959 cells, making them less suitable for modeling certain diseases like cancer. Finally, due to their substantial evolutionary divergence from man (Fig. 1), the targets of small molecules identified in invertebrates like *C. elegans* and *Drosophila* may not be conserved in man and even then the human orthologs may have divergent functions, making phenotypic screens using invertebrates less than ideal for drug discovery.

## 3 Zebrafish screens

We believe that zebrafish represent a “sweet spot” for large-scale phenotypic screens in terms of biological complexity, physiologic similarities to humans, small size and fecundity. Zebrafish are also far less costly to maintain in large numbers necessary to conduct a large-scale screen than mammals like mice. Although zebrafish have many important physiological differences from humans, they have numerous similarities such homologous organ systems and complex tissue architectures. Moreover, the majority of the functional domains of human proteins and zebrafish orthologs are highly conserved; many, if not a majority, of the small molecules discovered in zebrafish screens should have identical or closely related targets, in man. The first proof of principle that zebrafish could be useful for a large-scale *in vivo* phenotype screen came from a study that was conducted by Peterson and colleagues in 2000. In this study, the authors demonstrated that zebrafish embryos can be arrayed and screened in a 96-well format, and that small molecules which affected embryonic development and body patterning could be identified based on discrete perturbations to various anatomic structures [43]. Moreover, given the rapid development of zebrafish, which have a functional circulatory system by 24-hour post fertilization (hpf) and free swimming larvae by 72-hpf, the timeframe required for a phenotypic readout is similar to many cell based assays. Sixteen years since this landmark study, dozens, if not hundreds, of chemical screens have been carried out in zebrafish [45]. The phenotypic screens can be broadly be categorized into four major types by assay output: morphological, therapeutic, pathway and behavioral [66]. These four categories cover the majority of assays that have been performed in zebrafish and are meant to serve as a general framework for discussion of different assay types, rather than be comprehensive or mutually exclusive. Screens for compounds that modulate a diverse range of form and function, such as regeneration, lipid absorption and angiogenesis [45], while not specifically discussed here can be considered within the frame of the four categories.

### 3.1 Morphological

As the name indicates, the morphological screen involves identification of hit compounds based on their ability to cause specific and reproducible morphologic deviations from normal. The main feature of the morphology-based approach is the variable data depth of the screen, since they are by definition multi-dimensional [66]. The screener has the choice between obtaining “shallower data” by focusing exclusively on a single anatomical feature to “deeper data” to detect any discernable morphologic changes throughout the embryo. In a screen for compounds that result in altered dorsoventral (DV) patterning, we used tail length as a primary endpoint [72], since embryos with dorsalized pattern have grossly shortened, twisted tail [33]. This single point screen has resulted in the discovery of dorsomorphin, the first small molecule inhibitor of the bone morphogenetic protein (BMP) pathway [72] as well as a Wnt pathway modulator [13]. Even when focused on a single feature, the phenotypic screen can obtain additional information, increasing data depth. For example, Colanesi and colleagues performed a chemical screen looking specifically at the pigmentation of zebrafish embryo. From this simple phenotypic screen, they could subdivide the hit compound into 10 categories based on specific pigmentation alterations; these included reduced numbers of iridiophores and/or melanophores, changes in color depth in either cell type, ectopic numbers of chromatophores, abnormal shape of melanophores and so on [9].

Since the zebrafish embryo is transparent, the screener can simultaneously score for specific changes to a predefined morphologic feature and any morphologic changes in the rest of the body. We adopted this “all comer” approach to identify a novel hedgehog pathway inhibitor and a lysophosphatidic acid (LPA) receptor inhibitor [12], [52], [67], [68]. Importantly, because this screening approach is unbiased with respect to pathways and targets, it has the potential to allow discovery of novel mechanistic insights to regulation of pathways involved in embryonic development. Moreover, since it is open to all possible morphologic perturbations, the depth of phenomic data acquired is limited only by technology related to high-content image analysis. In addition, morphological screens are not limited to anatomical features visible by standard microscopy. For example, multiple groups have utilized transgenic fish expressing fluorescent markers in the endothelium to identify compounds that perturb the vasculature [47], [59]. Similarly, others have utilized transgenic fish expressing a fluorescent marker in cardiomyocytes to screen for compounds that effect both heart structure and function [5], [35]. It is also possible to conduct a fairly large-scale screen involving *in situ* hybridizations to screen for compounds that perturb expression patterns of a cell or tissue marker. For example, Zon and colleagues carried out an *in situ* hybridization-based screen to identify small molecules, such as leflunomide, which affect *crestin*-expressing neural crest cell development [65].

By definition, the morphology based screens are flexible, compatible with many derivations to discover small molecules that perturb many cell types and anatomical structures. Morphologic screens also serve as starting points for finding molecules that affect cell behaviors as well; for example looking at the quantity and location of leukocytes or neutrophils at a singular time point after tail resection provides information about where those cells are located, as seen by Liu et al., and Robertson et al. From here the authors used secondary assays to identify compounds that modulate the migration of these cells [28], [49]. An obvious shortcoming of the morphology-based screen is the lack of direct therapeutic relevance; nevertheless, the discovery of dorsomorphin by this approach has directly contributed to new therapeutic strategies for numerous human diseases such as heterotopic ossification, anemia, IBD, and cancers [15], [16], [17], [39], [40], [62] and has spawned several ongoing drug development programs.

### 3.2 Therapeutic

The therapeutic screen uses zebrafish with a disease phenotype to identify small molecules that specifically ameliorate this phenotype. In contrast to the morphological screen in which deviations from norm are the “hit” criteria, in this category, a return towards the normal phenotype would be a “hit”. In the first of such therapeutic screens in zebrafish, Peterson and colleagues used the *gridlock* mutant, a zebrafish model of aortic coarctation lacking normal tail circulation at 24 to 48-hpf, to identify small molecules which restored tail circulation [44]. Similarly, Peal and colleagues used the *breakdance* mutant, a zebrafish model of Long QT proarrhythmic syndrome due to a mutation in the KCNH2 potassium channel, to screen for compounds that ameliorate the proarrhythmic phenotype. In a relatively small screen of 1200 compounds, they identified two compounds that restored normal heart beating and therefore have potential as anti-arrhythmic agents [41]. In addition, other human disease models, such as Duchenne muscular dystrophy (DMD), have been successfully screened for compounds that suppressed the disease phenotypes [20].

Therapeutic screens have been successfully carried out in non-genetic disease models as well. Cardiomyopathy is a relatively common serious sequela of cancer treatment with the chemotherapeutic doxorubicin. Peterson and colleagues developed a zebrafish model of doxorubin-induced cardiomyopathy, and conducted a counter-screen for cardioprotective compounds [27]. Of the 3000 screened compounds, they discovered two, visnagin and diphenylurea, which protected cardiac function without mitigating the chemotherapeutic effects. In a similar manner, the Peterson group also screened for chemoprotectors against cyanide poisoning, and identified four potential antidotes [34]. In a search for candidate compounds that can accelerate recovery after acute kidney injury (AK), Cianciolo Cosentino et al. screened for small molecules that increase proliferation of renal progenitor cells in zebrafish embryos [6]. This screen identified histone deacetylase inhibitor methyl-4-(phenylthio)butanoate (PTBA), which enhanced recovery after acute kidney injury [6] and reduced postinjury renal fibrosis in mice [54]. Finally, investigators have developed *Mycobacterium marinum* infection and human carcinoma xenograft models in zebrafish [19], [57]. These two models allow for identification of compound that selectively kill pathogen or tumor cells without affecting the health and viability of zebrafish. The paradigm of therapeutic screening in zebrafish is attractive because of its immediate therapeutic relevance. While such screens show promise, using the correct model for screening is critically important to ensure the validity of the therapeutic target. With the ease of genetic editing through CRISPR/Cas9, this platform would be particularly well suited for monogenic diseases with well understood pathophysiology, as zebrafish based models could be rapidly developed and screened.

### 3.3 Pathway

The pathway screen involves identification of hit compounds based on their ability to perturb the function of a specific pathway of interest. As with other phenotypic screens, the assay is unbiased with respect to a particular molecular target; however, it limits the scope of potential targets as the hit must interact with a specific pathway in a measurable manner. This modality relies on pathway-specific read outs in the zebrafish. One of the first pathway screens in zebrafish was conducted by Molina and colleagues. In this study, the authors took advantage of the fact that gene expression of *dual specific phosphatase-6* (*dusp6*), a feedback regulator of FGF (fibroblast growth factor) signaling, is itself a robust reporter of FGF pathway activations. They used a transgenic zebrafish expressing a destabilized GFP expressed under the control of a dusp6 promoter. In this platform, the GFP fluorescence intensity provides quantitative read out of the signaling activity [30]. From this screen, Molina and colleagues identified a compound (E)-2-benzylidene-3-(cyclohexylamino)-2,3-dihydro-1H-inden-1-one (BCI), and used chemical genetic epistasis and computational approaches to show that the compound targeted dusp6 itself. One drawback of GFP reporters in zebrafish is that quantification of fluorescence can be difficult given the dynamic nature of the transgene expression pattern and that the orientation of the zebrafish in a well can dramatically affect the apparent signal intensive. To address some of these issues, *in vivo* luciferase reporter fish lines have been developed [1], [64].

Finally, a single assay could have a combination of morphological, therapeutic and pathway outputs For example, in the *axin* mutant embryos, ectopic activation of the canonical Wnt/β-catenin signaling results in an eyeless phenotype [63], and the *axin* mutant phenotype can be recapitulated with BIO, an inhibitor of GSK3β, a key component of the β-catenin destruction complex inhibitor. Moreover, windorphen, a canonical Wnt pathway inhibitor, can rescue the eyeless phenotype in *axin* mutants [13]. Using this Wnt pathway-specific morphologic phenotype as a read out, Nishiya and colleagues conducted a chemical suppression screen and discovered that GGTI-286, a geranylgeranyltransferase 1 (GGTase I) inhibitor, could block canonical Wnt signaling downstream of the β-catenin destruction complex [36]. In the developing zebrafish embryo, individual pathways do not exist in isolation; therefore, a phenotypic screen designed to interrogate one signaling pathway may lead to serendipitous and sometimes context specific interaction with other pathways.

### 3.4 Behavioral

One of the major unmet therapeutic areas is in neuropsychiatric diseases, for which many target based drug discovery efforts have failed. Such difficulties and the fact that many current neuropsychiatric medicines trace their roots to clinical observations on neurobehavioral effects of drugs originally intended for other indications have motivated investigators to consider behavioral screens to discover novel, and hopefully physiologically relevant, neuropsychiatric drug targets. For this, zebrafish larvae seem ideal since they are amenable to high-throughput chemical screens and they exhibit numerous complex behaviors reminiscent of some human behaviors. In one of the first behavior-based chemical screen in zebrafish, Rihel and colleagues screened over 5000 compounds for modulators of restfulness or wakefulness. This screen resulted in the identification of 463 unique structures that altered zebrafish behavior [48]. Of these compounds, known modulators of major neurotransmitters were found to recapitulate many of the behavioral effects observed in mammals. For example clonidine, a α2-adrenergic receptor agonists used as a treatment for ADHD (attention deficit hyperactivity disorder) and gaining use as a sedative, was found to also have sedating effects in zebrafish. Subsequent behavioral screens for compounds that modulate responses to photic and acoustic stimuli yielded compounds that not only modulate immediate responses to these stimuli, but also more complex behaviors such as habituation [22], [69]. This technology has been expanded to a battery of tests to identify novel neuroactive compounds with a distinct behavioral profile, a “fingerprint,” which can then be used to inform mechanism of action studies [3], [46]. With ongoing advances in behavioral analysis algorithms, it may one day be possible to screen for compounds that modulate increasingly complex behaviors. Given difficulties in developing drugs for neuropsychiatric diseases by targeted approaches, zebrafish-based behavioral screens represent a bold new path for this important unmet medical need as well as opportunities to improve our understanding of animal behavior.

## 4 Beyond discovery

While still relatively new, the impact of zebrafish-based chemical screens has been notable. In the past decade, the rate of published zebrafish screens has risen steadily, with an average impact factor of 9.5, as of 2013 [45]. While dissemination of knowledge through the publication of a chemical screen is the primary goal for academics, a secondary, implicit goal is therapeutic discovery, ultimately to impact human health. Among a number of compounds originally identified in zebrafish chemical screens with therapeutic potential, several have resulted in industry partnerships for preclinical and clinical development. For instance, Oricula Therapeutics is developing Proto-1 for prevention of hearing loss, Novo Biosciences is developing a metalloproteinase-13 (MMP13) inhibitor for peripheral neuropathy, and La Jolla Pharmaceuticals is developing BMP receptor inhibitors for fibrodysplasia ossificans progressiva (FOP) and other rare diseases. The most advanced therapeutic lead resulting from a zebrafish screen is the PGE2 inhibitor Prohema [38], which has shown promising results in a randomized, controlled Phase II study of patients undergoing hematopoietic stem cell (HSC) transplantation for the treatment of hematologic malignancies [11]. Given these early successes, it seems reasonable to anticipate that there will be many more therapeutic leads resulting from zebrafish chemical screens in the coming decades.

## 5 Next steps: Genomics and drug discovery

**Fig. 2:**
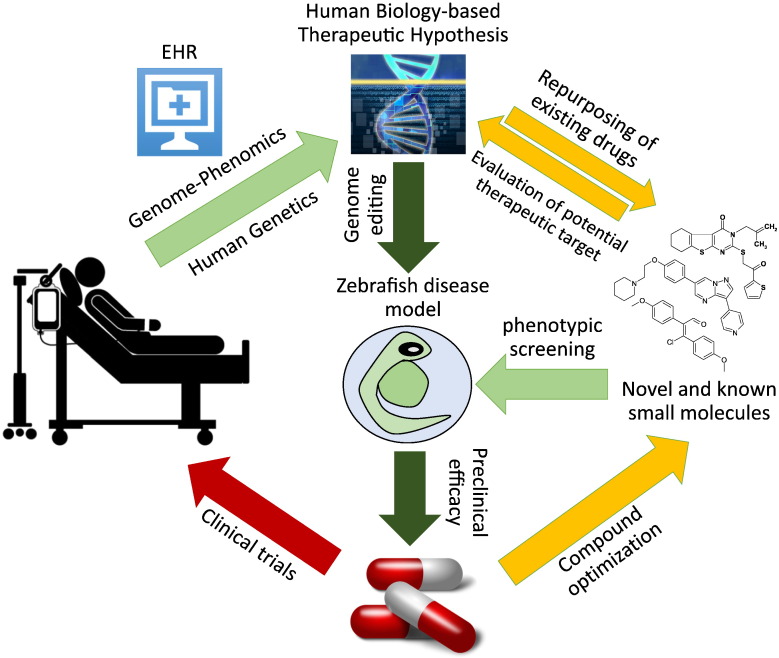
Proposed zebrafish phenotypic screens incorporating human genome–phenome information to accelerate therapeutic discovery. Human genome–phenome information provided by electronic health record (EHR)-coupled DNA database and by human genetic diseases studies drive formulation of therapeutic hypotheses (“human biology-based therapeutic hypotheses”). To test these hypotheses, zebrafish models of human genetic diseases are generated by genomic editing and employed in phenotypic screen for novel or known compounds which ameliorate the disease phenotypes. These compounds are then advanced for further development, including compound optimization and testing in appropriate preclinical disease models. Alternatively, a target-agnostic morphology-based screen is carried out. Subsequently, targets of hit compounds identified, and each target evaluated *in silico* against human genome–phenome database to determine whether a viable therapeutic hypothesis can be formulated. If so, these hits are advanced for further development, including compound optimization and testing in appropriate preclinical disease models.

There are currently about 7000 known rare diseases in man, and roughly 4000 of these have been linked to a single genetic cause [26], [55]. Some, like familial hypercholesterolemia, are fairly common, found in 1 in 500 individuals, while other are extremely rare like fibrodysplasia ossificans progressiva (FOP), found in 1 in 2 million individuals. Taken together, about 10% of the US population is estimated to be afflicted with a rare disease, representing a significant healthcare burden [14]. Of the disease associated genes in the Online Mendelian Inheritance in Man (OMIM) database, 82% have at least one zebrafish ortholog [18]. With the advances in genome editing technology, such as the clustered regularly interspaced short palindromic repeats CRISPR/Cas9 nuclease technique, it is now feasible to generate zebrafish models of virtually all human Mendelian diseases (Fig. 2). Once a disease phenotype or a surrogate phenotype is established in zebrafish mutants, a therapeutic screen for compounds that ameliorate these phenotypes should be straightforward. With the advances in genomic sequencing technologies, the number of ultra-rare genetic diseases is expected to increase significantly in the coming decade. In such a scenario, one can easily envision harnessing the power of zebrafish phenotypic screens, perhaps using a panel of known bioactive small molecules or FDA approved drugs, to help accelerate drug discovery and repurposing efforts for rare genetic diseases (Fig. 2).

A unique advantage of phenotypic screens is the discovery of novel, previously unrecognized components involved in a biological process or a disease pathophysiology, and chemical tools to modulate them. However, the discovery of new pharmacological targets and new pharmacological classes by themselves do not ameliorate the most important reason for the high rate of failure in drug development: uncertainties associated with target selection. Based on the first principles, the risks associated with target selection are inherently lower for human Mendelian conditions. For instance, the knowledge that rare individuals lacking proprotein convertase subtilisin/kexin type 9 (PCSK9) have better lipid profiles and are protected from atherosclerosis and myocardial infarctions was an important factor in rapid development and approval of PCSK9 inhibitor for treatment of hypercholesterolemia [7], [8], [23], [73]. But the power of human genetics need not stop with rare Mendelian conditions. At Vanderbilt University Medical Center (VUMC), a large human DNA repository, named BioVU, has been linked to de-identified electronic health records (EHR) within the Synthetic Derivative (SD) database. Using BioVU as a human genome–phenome analysis platform, a phenome-wide association study (PheWAS) can be carried out to determine what clinical phenotypes are associated with single nucleotide polymorphisms (SNPs) in a given gene (Fig. 2;[10]). Using this approach, we not only identified potential new indications of our small molecule BMP inhibitors but also potential on-target side effects, which will be valuable for eventual clinical trials and post-marketing surveillance (CCH, personal communication).

How might zebrafish-based phenotypic screens leverage the power of human genetics to accelerate drug discovery? As discussed above, zebrafish models of human Mendelian genetics can be used to carry out therapeutic screens for compounds that ameliorate the disease phenotype (Fig. 2). Alternatively, a novel pharmacological target identified in unbiased morphologic screens can be interrogated by phenome–genome databases such as BioVU to determine whether alterations in that gene are associated with a disease phenotype and/or therapeutic effects, dramatically lowering the risks of a drug development program (Fig. 2). If carried out on a large scale, such efforts might dramatically accelerate drug discovery and repurposing efforts to meet the anticipated need for targeted therapies for rare and common diseases (Fig. 2). In summary, zebrafish is a versatile platform that has a bright future as a drug discovery tool in the Era of Personalized Medicines.

## Disclosure

C.C.H. holds patents and patent applications on composition of matter and methods of use of small molecule BMP inhibitors.
